# Oxidative Stress Level in the Testes of Mice and Rats during Nickel Intoxication

**DOI:** 10.1100/2012/395741

**Published:** 2012-02-01

**Authors:** Eugenia Murawska-Ciałowicz, Wojciech Bal, Lidia Januszewska, Marcin Zawadzki, Joanna Rychel, Jolanta Zuwała-Jagiełło

**Affiliations:** ^1^Physiology and Biochemistry Department, University of Physical Education, Avenue I.J. Paderewskiego 35, 51-612 Wroclaw, Poland; ^2^Hygiene Department, Medical University, Ul. J. Mikulicza-Radeckiego 7, 50-368 Wroclaw, Poland; ^3^Biochemistry and Biophysic Institute, Polish Academy of Sciences, Ul. Pawińskiego 5a, 02-106 Warsaw, Poland; ^4^Forensic Medicine Department, Medical University, Ul. Mikulicza-Radeckiego 4, 50-368 Wroclaw, Poland; ^5^Pharmaceutical Biochemistry Department, Medical University, ul. Szewska 38/39, 50-139 Wroclaw, Poland

## Abstract

The genotioxic and carcinogenic effect of nickel probably results from its capacity to produce reactive oxygen species (ROS) and disturb the redox balance. The aim of the study was to find out if rats lacking spermatic protamine 2 are less susceptible to Ni(II) than mice. Consequently, the levels of malondialdehyde + 4 hydroxynonenal (MDA+4HDA) − markers of lipid peroxidation, as well as the level of reduced glutathione (GSH) were measured within the rat and mouse testes. Our results showed that the levels of lipid peroxidation markers were elevated in testicular homogenates of intoxicated mice without any changes in rats. GSH level was lower in the group of intoxicated mice comparing to the control without statistically significant changes in rats' homogenates. Moreover, the level of GSH in the testes of intoxicated mice was lower than in rats. On the basis of our results, it appears that Ni(II) can initiate oxidative stress in the testes of mice but not of rats and can reduce GSH level. Consequently, the antioxidative defense of the testes is reduced. Ni(II) that causes oxidative stress in the testes may also contribute to infertility.

## 1. Introduction

Nickel is a heavy metal present in all elements of the environment. It is the fifth most widespread element on Earth. In chemical compounds, it has a few oxidation states, −1 being the lowest and +4 the highest. However, the most common state of nickel is +2 [Ni(II)] [1].

Ni(II) compounds are used in different industries and for producing everyday objects. It can be used in shipbuilding, chemical, electrochemical, and galvanizing industries. It is used for producing Ni-Cd batteries, stainless steel, bathroom fittings, coins, colourings, kitchenware, cutlery, surgical instruments, dental and orthopedic prostheses, artificial jewellery, and so forth. Thus, nickel exposure is a problem of the whole population and allergies to nickel are reported quite often (10% women, 1% men) [2, 3].

Nickel usually enters the body via inhalation, ingestion, and dermal absorption. Professional exposure concerns people whose jobs are connected with nickel extraction and processing. These include refinery workers, galvanizers, welders, chemists, and jewelers. In this kind of exposure, the most important permeation route of Ni(II) into the body is inhalation and dermal absorption [1, 4–6].

 As far as the general population is concerned, the most important exposure is the dermal absorption. In that way, nickel penetration into the body can be constant because people are in contact with nickel products on a daily basis [7]. As far as inhalation is concerned, the level of absorption depends on the chemical form of nickel present in the food. The material that kitchenware is made of is also of significance [1]. However, most of the nickel absorbed this way is subject to elimination.

After being absorbed into the bloodstream, nickel is transported by albumin. It is accumulated mainly in kidneys, the liver, lungs, and testes [7–9]. Ni(II) is a human carcinogen. Powder nickel and its salts belong to group 1 carcinogens [3]. Epidemiological evidence shows high incidence of nasal and respiratory track cancers in miners and refinery workers [4, 7].

A very important aspect of Ni(II) toxicity is also the fact that it crosses the placenta barrier and the blood-testis barrier, which is important for fertility and proper development of embryos [10]. The molecular mechanism of genotoxic activity of nickel compounds and mechanisms at the bottom of male infertility are not fully explained. The theory of free radicals can constitute part of the explanation of this activity [3, 11]. Free radicals are produced, among others, in the Fenton reaction which requires the presence of H_2_O_2_ and [Fe(II), Cu(II)] ions. The product of the reaction is a very reactive hydroxyl radical (OH^•−^), which reacts with all molecules in its surroundings. It damages, among others, nucleic acids causing the oxidation of nucleic bases, ribose, and deoxyribose. It has been confirmed by model research in which an increase in the concentration of 8-hydroxy-2′-deoxyguanosine (8-OH-dG) was observed [1, 12–14]. This product is probably a promutagen and might cause the appearance of carcinogenic changes in children whose fathers were exposed to nickel compounds (before their children were conceived) [15].

One of the effects of free radical reactions is the replacement of other metals with nickel in binding sites. Protamine 2 (P2) has become the “target” of the nickel attack. It is a protein indispensable for the production and maturation of sperm in mammal cells. It is possible due to the specific structure of protamine 2 which allows its binding with zinc. However, it is possible to replace zinc with ions of other metals, for example, Ni(II) [5]. It has been found that the nickel-protamine P2 interaction prevented normal chromatin condensation. The lowered level of the protein is most likely the reason for male infertility, and oxidative activity of Ni(II) increases its interaction with P2 thus leading to changes in the DNA structure and the appearance of oxidation products, which are promutagens.

Research into human lymphocytes showed that oxidative changes to DNA are connected with a decrease in the level of reduced glutathione (GSH)—an important intracellular radical that directly or indirectly reacts with free radicals thus taking part in detoxification reactions.

Taking into account the fact that free radical generation is the basis of the genotoxic effect of Ni(II) and knowing that glutathione contributes to the reduction of damage to DNA, the primary aim of the study was to conclude whether male infertility caused by exposure to Ni(II) may be a result of oxidative stress affecting protamine 2 and whether rats being animals lacking protamine 2 are less sensitive to Ni(II), contrary to mice, which have that protein.

## 2. Materials and Methods

### 2.1. Chemicals

Nickel chloride was obtained from Sigma Chemicals Co. (St. Louis, USA), and reagent kits from Calbiochem (La Jolla, CA, USA) and OXIS (OXIS International Inc., USA).

### 2.2. Experimental Protocol

The studies were performed on 25 male rats (*R*) of the Buffalo strain, weighing 200–250 g and on 25 male mice (*M*) of the Balb/c strain, weighing 30–40 g. The animals were kept in conditions consistent with requirements of the local commission for ethical matters of animal experimentation. They were maintained in controlled environmental conditions of ambient temperature (22 ± 1°C) and relative humidity of 40–60%, in a 12 : 12 light/dark cycle. All animals were fed standard pelleted diet and water ad libitum. The animals were divided into four groups: 10 rats and 10 mice in the controls (*C*) and 15 rats and 15 mice in the intoxicated (*I*) groups. The control groups received i.p. injections of 0.9% NaCl once. Animals intoxicated (*I*) with Ni(II) received once an i.p. injection of 5 mg Ni(II)/kg b.w. in the form of NiCl_2_.

48 hours after intoxication, the animals were put to sleep with ketamine injection of 100 mg/kg m.c. and their cervical vertebrae were dislocated. Afterwards their testes were removed for further research. They were weighed and frozen at the temperature of −86°C.

### 2.3. Biochemical Analysis

For biochemical tests, the samples were thawed, washed with 0.9% NaCl plus EDTA, homogenized in ice-cold 20 mM Tris-HCl Buffet (pH = 7.4), and centrifuged for 10 min at the temperature of 4°C at 15 000 rpm. The supernatants were decanted and used to estimate markers of lipid peroxidation − malonylodialdehyde + 4hydroxynonenal (MDA + 4HDA), levels using Lipid Peroxidation Assay Kit (Calbiochem) and of reduced glutathione (GSH) using Bioxytech GSH-400 kit (OXIS).

### 2.4. Statistical Analysis

Statistical analysis was performed using the Statistica 9.1 PL software (StatSoft, Cracow, Poland). The significance of differences between control animals and intoxicated animals was tested employing the nonparametric Mann-Whitney *U* test at the confidence level of *P* < 0.05.

## 3. Results

As shown in Table 1, MDA + 4HDA concentration in testicular homogenates of mice in the control group was almost twice as high as the concentration recorded in the rats control groups. In the groups of intoxicated rats, the concentration of lipid peroxidation markers did not increase after exposure to Ni(II), whereas in the group of mice there was a significant increase in MDA + 4HDA as a result of Ni(II) intoxication (by 25%) and in comparison with intoxicated rats (*P* < 0.05).

**Figure 1:**
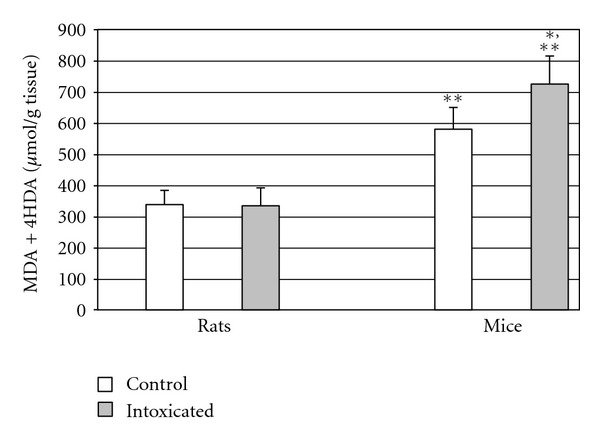
Concentration of lipid peroxidation markers in testicular homogenates of mice and rats. *P < 0.05 in comparison to the control, **P < 0.001 in comparison to both groups of rats.

**Figure 2:**
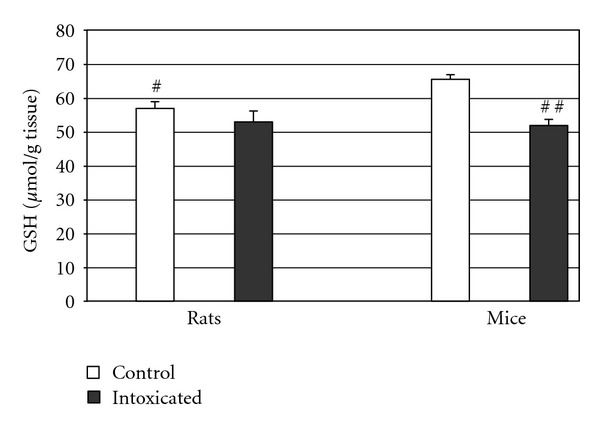
Concentration of reduced glutathione in testicular homogenates of rats and mice, ^#^ *P* = 0.05 in comparison to the mice control, ^##^ *P* = 0.0029 in comparison to the control.

As shown in Table 1 and Figure 1, mice exposed to Ni(II) turned out to have an over twofold increase in the level of lipid peroxidation markers (*P* < 0.001).  Table 2 and Figure 2 show that GSH concentration in testicular homogenates of intoxicated rats was not significantly affected by Ni(II). In the group of intoxicated mice, the concentration of this antioxidant was significantly lowered (by 20%, *P* < 0.005). GSH concentration in the control group of mice was significantly higher in comparison to the control group of rats. In intoxicated groups, there were no differences in GSH concentration.

## 4. Discussion

It has been shown in the present study that during exposure to nickel there is a significant increase in lipid peroxidation in testicular homogenates of animals that have protamine 2. In the group of rats exposed to Ni(II), concentration of peroxidation markers in testicular homogenates did not change. This suggests that mice are more sensitive to the activity of the nickel ions than rats. The findings are consistent with the research presented by Belokopytowa [16], according to which rats have hardly any protamine 2 in comparison with mice. Protamine P2 in rats accounts for only 2–5% of the amount found in mice. Unlike P2, protamine 1 was found in sperm nuclei of all mammal species. P2 was only found in sperm of men, horses, mice, and hamsters [17, 18]. Most likely it is the main protein binding zinc and stabilizing the sperm chromatin and it is essential for male fertility [19]. While looking for reasons for male infertility, it was concluded that the most likely cause is the lowered level of human protamine 2 in sperm [16]. While analyzing the ratio of protamine P1 and P2 in the semen of fertile male mice and hamsters and in the semen of a man, it was concluded that the ratio of P1 : P2 is constant for a given mammal species but it differs a lot between species. In a man, the ratio is 1 : 1. Damage to protamine or a changed P1/P2 ratio results in fertility reduction [20, 21].

On the basis of our results, it appears that rats are less sensitive to oxidative stress caused by nickel ions; therefore, damage done to the DNA of their sperm should be smaller in comparison with DNA damage in mice, which might make their survival in difficult environmental conditions easier. An increase in lipid peroxidation in mouse testes has also been observed in other studies [8, 22] which examined a few generations of Ni(II) intoxicated mice. The fertility of the mice deteriorated from generation to generation.

Genetic material stored in sperm is a very probable target of the Ni(II) attack [1]. It has been confirmed in the present research, as well as in the experiment in which a group of male mice was exposed to NiCl_2_. A temporary accumulation of Ni(II) occurred in the males' testes [22, 23]. It was accompanied by chromosome aberration, a decrease in the number of sperm, and a visible increase in reactive oxygen species. Damage to DNA by reactive oxygen species consists in, among others, changes in nucleic bases, production of intrastrand cross links between nucleic bases, production of cross links between DNA chains, splitting of a DNA chain, and depurination (removal of purine bases). That damage makes the passage of correct genetic information impossible [24].

Protamines can modulate DNA damage. Heavy metals, and Ni(II) especially, can be attached to protamine 2. This peptide has a characteristic sequence of the Arg-Thr-His amino acids at their N-terminus. This sequence can be a “trap” for heavy metals. The Ni(II) binding to protamine 2 is very strong and specific [25, 26]. To examine in a more detailed way the toxicity of Ni(II) after its binding to protamine 2, Bal and his team [25, 26] synthetized a peptide made up of 15 amino acids (HP_1–15_), which at the N-terminus had a sequence of amino acids identical with human protamine 2. It was noticed that HP2_1–15_ binds Ni(II) very strongly. The HP2_1–15_-Ni(II) complex created as a result of the binding was able to activate oxygen and H_2_O_2_. Additionally, that complex was more closely related to DNA than the HP2_1–15_ itself. After being bound with HP2_1–15_, nickel was activated and it became an agent of DNA splitting by H_2_O_2_. It was also concluded that the HP2_1–15_-Ni(II) complex led to the production of radical forms such as OH^•-^ and superoxide O_2_^•−^.

Spermatids are rich in mitochondria. As a result of intensive oxygen metabolism, they can “leak” H_2_O_2_. Metal-binding protamine 2 is present in sperm, too. If an organism is exposed to nickel compounds, nickel binds to protamine 2 in sperm, and reactive oxygen species are created from H_2_O_2_. Sperm death is one of the consequences of this oxidization [24, 27].

 In the present study, changes in GSH concentrations in rats' and mice's testes after the application of nickel compounds were measured. It was noticed that GSH concentration in the testes after the exposure to Ni(II) decreases in the mice group, with no significant changes in the rats group. From our results it can be concluded that healthy mice's testes are characterized by significantly higher GSH concentration in comparison with the rats. It might point to the fact that rat testes, less sensitive to activities of free radicals, do not have to be characterized by antioxidative potential as big as mouse testes. Similarly, Li et al. [28] proved that the application of Ni(II) results in a decrease in glutathione level. Krążel et al. [29] found out, *in vitro*, that GSH can hamper oxidative influence of Ni(II) on DNA, in concentration much exceeding the amount of Ni(II) in the environment. Tremellen [30] claims that a decrease in GSH concentration indispensable for the maintenance of GPx activity creates conditions for the disturbance of redox homeostasis of the semen exposing it to oxidative damage.

 Other authors have also carried out interesting research [31–35]. They measured total ability of antioxidative seminal plasma of fertile and infertile men. The researchers noted that the total antioxidative level in samples obtained from infertile men was significantly lower. While examining the amount of Zn in seminal plasma, Colagar et al. [36] found lower concentration in infertile men. Zn also has antioxidative potential and participates in ROS scavenging.

Reduced activity of antioxidants leads to an increase in DNA damage, changes in sperm morphology, and a reduction in sperm motility. In all of the above-mentioned studies, researchers recorded a decrease in the concentration of intracellular antioxidants, and as a consequence, a reduced ability of cells to protect and regenerate themselves. Ni(II) was one of the numerous damaging factors. Because of the ability of nickel ions to penetrate the blood-testis barrier, to easily bind to protamine 2, and to mediate in production of free radicals, their presence in the testes leads to male infertility. Cocuzza et al. [37] think that an increase in the generation of ROS causes the lowering of semen quality, a decrease in the amount of sperm, and a reduction in sperm motility. At the same time they emphasize that leukocytes are an additional factor that increases the generation of ROS in semen. The number of leukocytes can increase as a result of an inflammatory process initiated by Ni(II). Recently it has been shown that apart from protamine 2, there is another target for a Ni(II) attack and it is glutamate-ammonia ligase (GLUL) that is more closely related to Ni(II) than to its regular cofactor manganese [38].

On the basis of the results obtained from our research, it can be stated that the mechanism of toxic activity of Ni(II) in the testes is oxidation-based. This is the first research that indicates the differences in the level of stress in animals that either have or lack protamine 2. In case of exposure to Ni(II), ROS, which are responsible for DNA damage, are produced in the testes.

## Figures and Tables

**Table 1:** MDA + 4HDA concentration (*μ*mol/g of the tissue) in groups of intoxicated rats and mice and in control groups.

Groups	Controls (*C*)	Intoxicated groups (*I*)	Significance *C* : *I*
Rats (*R*)	339.62 ± 28.29	333.52 ± 54.69	*n.s*
Mice (*M*)	579.8 ± 76.02	724.70 ± 90.74	*P < 0.05 *
Significance *R* : *M*	*P < 0.001 *	*P < 0.001 *	

**Table 2:** GSH concentration (*μ*mol/g of the tissue) in the groups of intoxicated rats and mice and in the control groups.

Groups	Controls (*C*)	Intoxicated groups (*I*)	Significance *C* : *I*
Rats (*R*)	56.92 ± 2.96	52.88 ± 3.25	*n.s*
Mice (*M*)	65.46 ± 1.35	51.80 ± 1.85	*P = 0.0029 *
Significance *R* : *M*	*P = 0.05 *	*n.s *	
